# Discordance in Tumor Mutation Burden from Blood and Tissue Affects Association with Response to Immune Checkpoint Inhibition in Real-World Settings

**DOI:** 10.1093/oncolo/oyab064

**Published:** 2022-02-19

**Authors:** Emma G Sturgill, Amanda Misch, Carissa C Jones, Daniel Luckett, Xiaotong Fu, Dan Schlauch, Suzanne F Jones, Howard A Burris, David R Spigel, Andrew J McKenzie

**Affiliations:** Sarah Cannon Research Institute, Nashville, TN, USA; Sarah Cannon Research Institute, Nashville, TN, USA; Genospace, Boston, MA, USA; Sarah Cannon Research Institute, Nashville, TN, USA; Sarah Cannon Research Institute, Nashville, TN, USA; Genospace, Boston, MA, USA; Sarah Cannon Research Institute, Nashville, TN, USA; Genospace, Boston, MA, USA; Sarah Cannon Research Institute, Nashville, TN, USA; Genospace, Boston, MA, USA; Sarah Cannon Research Institute, Nashville, TN, USA; Sarah Cannon Research Institute, Nashville, TN, USA; Tennessee Oncology, Nashville, TN, USA; Sarah Cannon Research Institute, Nashville, TN, USA; Tennessee Oncology, Nashville, TN, USA; Sarah Cannon Research Institute, Nashville, TN, USA

## Abstract

**Background:**

Tumor mutation burden (TMB), a biomarker for immune checkpoint inhibitor (CPI) response, is reported by both blood- and tissue-based next-generation sequencing (NGS) vendors. However, the agreement between TMB from blood (bTMB) and tissue (tTMB) in real-world settings, both in absolute value and association with CPI response, is not known.

**Materials and Methods:**

This study utilizes Sarah Cannon’s precision medicine platform, Genospace, to harmonize clinico-genomic data from 17 206 patients with cancer with NGS results from September 2015 to August 2021. A subset of patients have both bTMB and tTMB results. Statistical analyses are performed in R and include (1) correlation (*r*) and concordance (ρ) between patient-matched bTMB-tTMB pairs, (2) distribution of total bTMB and tTMB values, and (3) association of bTMB and tTMB with time to CPI therapy failure.

**Results:**

In 410 patient-matched bTMB-tTMB pairs, the median bTMB (*m* = 10.5 mut/Mb) was significantly higher than the median tTMB (*m* = 6.0 mut/Mb, *P* < .001) leading to conflicting “high” and “low” statuses in over one-third of cases at a threshold of 10 mut/Mb (*n* = 410). Significant differences were observed in the distribution of bTMB values from blood-NGS vendors, with guardant health (GH) reporting higher (*m* = 10.5 mut/Mb, *n* = 2183) than Foundation Medicine (FMI, *m* = 3.8 mut/Mb, *n* = 462, *P* < .001). bTMB from GH required a higher threshold (≥40 mut/Mb) than bTMB from FMI (≥12 mut/Mb) in order to be associated with CPI response.

**Conclusions:**

This study uncovers variability in bTMB reporting among commercial NGS platforms, thereby evidencing a need for assay-specific thresholds in identifying patients who may respond to CPI therapy.

## Teaser

Identifying those patients who will benefit from immune checkpoint inhibitor therapy is a major challenge. This article assesses the concordance between blood tumor mutation burden (bTMB) and tissue tumor mutation burden (tTMB) from patients in real-world settings and compares associations with immune checkpoint inhibitor response.

**Implications for Practice**

In patients with both blood- and tissue-based sequencing results, the median tumor mutation burden (TMB) is higher in blood (10.5 mutations per megabase) than in tissue (6.0 mutations per megabase). This discordance necessitates different thresholds for TMB from blood (≥40 mutations per megabase) and tissue (≥10 mutations per megabase) to be associated with response to immune checkpoint inhibition. A higher TMB threshold should be considered for certain commercial blood-based next-generation sequencing tests when determining potential benefit from immune checkpoint inhibition.

## Introduction

To evade detection by a host’s immune system, cancer cells trigger immune checkpoints that signal through cell surface proteins including PD-1/PD-L1 and CTLA-4.^1^ Antibodies that mask these cell surface proteins function as immune checkpoint inhibitors (CPIs) and are used to treat cancer.^2^ The clinical development of CPIs has rapidly expanded over the past 10 years with many CPIs now integrated into standard-of-care across many cancer types.^3^ Despite these successes, a prominent challenge remains identifying which patients will benefit from CPI therapy.^4^ A key objective of the immuno-oncology field is to develop CPI predictive biomarkers in order to stratify patients into potential responders and non-responders.^5^

Emerging CPI biomarkers can broadly be categorized into those that report on inflammation in the tumor microenvironment and those that report on neoantigen load.^6^ The latter refers to the abundance of non-self peptides arising from mutations in the tumor genome and has been demonstrated to correlate with response to CPI therapy.^7^ In clinical settings, tumor mutation burden (TMB) is used as a proxy for neoantigen load in predicting potential benefit from CPI therapy.^6,7^ Generally defined as the number of somatic mutations within the tumor genome, TMB can range up to hundreds of mutations per megabase (mut/Mb) with distributions varying across cancer types.^8^

While TMB is most accurately measured by whole-exome sequencing, it can be estimated by targeted NGS panels in clinical settings.^9,10^ The Foundation Medicine (FMI) and Memorial Sloan Kettering (MSK)-IMPACT NGS assays were among the first to demonstrate the predictive ability of TMB, with the Food and Drug Administration ultimately approving FMI’s FoundationOne CDx assay as a companion diagnostic for pembrolizumab in High-TMB (ie, ≥10 mut/Mb) solid tumors.^9,11-15^ Subsequent studies have shown that TMB estimation can be impacted by a number of key variables including gene panel size, sequencing depth, variant callers, and filters,^14^ such that clinicians must use caution when extending TMB estimation to routine practice. Efforts to standardize TMB reporting are underway in order improve consistency and reliability across NGS platforms and aid in clinical decision making.^16^

Pre-analytical factors concerning specimen preparation (eg, fixation methodology, tumor purity, etc.) also impact TMB estimation.^14^ With the recent advent of TMB reporting by blood-NGS assays,^17-19^ it has become imperative to understand how TMB from blood (bTMB) and tissue (tTMB) compare. Previous studies have described a positive correlation between patient-matched bTMB and tTMB values^20,21^; however, the overall agreement and clinical validity of bTMB have to our knowledge not been examined. In this study, we assess the concordance between bTMB and tTMB from patients in real-world settings and compare their association with CPI response. Our results demonstrate that assay-specific thresholds must be implemented for bTMB to predict CPI response, thereby cautioning against a blanket 10 mut/Mb threshold for stratifying potential CPI responders in the clinic.

## Methods

**Figure 1.**
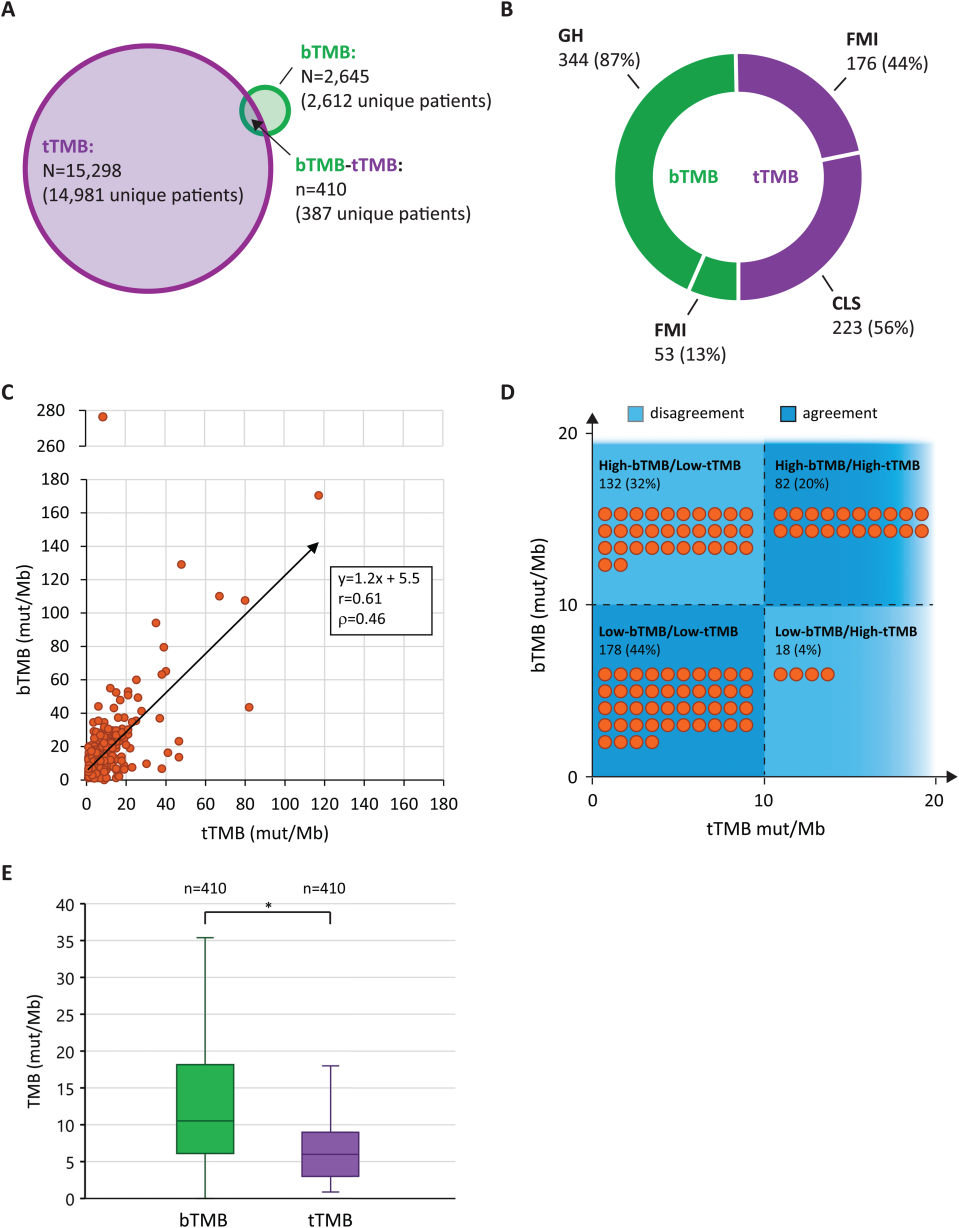
Discordance in patient-matched bTMB and tTMB values. (**A**) Overview of dataset showing number of tTMB (purple) and bTMB (green) values assessed. Number of unique patients are also indicated (note that patients with repeat sequencing can be associated with more than one TMB value). Overlap depicts number of patient-matched bTMB-tTMB pairs. (**B**) Breakdown of patient-matched bTMB (green) and tTMB (purple) values by NGS vendor. (**C**) Scatter plot of patient-matched bTMB-tTMB pairs. Linear trendline, Pearson’s correlation coefficient (*r*), and Lin’s concordance coefficient (ρ) are shown. *n* = 410. (**D**) Distribution of bTMB-tTMB pairs into high and low categorical statuses based on a threshold of 10 mut/Mb. Each orange circle represents 1% of bTMB-tTMB pairs. Quadrant color depicts agreement (dark blue) and disagreement (light blue) in bTMB and tTMB status. *n* = 410. **(E)** Distribution of bTMB (green) and tTMB (purple) values from patient-matched pairs. bTMB: *m* = 10.5 mut/Mb; IQR = 12.1 mut/Mb; *n* = 410. tTMB: *m* = 6.0 mut/Mb; IQR = 6.0 mut/Mb; *n* = 410. ∗*P* < .001. NGS, next-generation sequencing. Mut/Mb, mutations per megabase. m, median. IQR, interquartile range. GH, guardant health. FMI, Foundation Medicine. CLS, Caris Life Sciences. n.s., not significant.

Using a precision medicine platform to harmonize clinical and genomic data, we assessed 410 patient-matched bTMB-tTMB pairs from 387 unique patients (the number of bTMB-tTMB pairs exceeds the number of unique patients because a subset of patients had multiple blood- and/or tissue-NGS reports; Fig. 1A). Tissue-NGS tests (*n* = 399) were conducted by Caris Life Sciences^22^ (CLS; 56%) and FMI^8^ (44%; Fig. 1B). Blood-NGS tests (*n* = 397) were conducted by GH^23^ (87%) and FMI^18^ (13%; Fig. 1B). Clinical data were from 73 clinics across the Sarah Cannon network.

### Patient Population

Data were analyzed from 17 206 unique patients with TMB results from blood- and/or tissue-NGS testing. A range of solid tumor diagnoses as determined by the submitted diagnosis field on the NGS reports were represented. Of the 17 206 patients, 387 had TMB values from both blood- and tissue-NGS, from which we derived 410 patient-matched bTMB-tTMB pairs (Fig. 1A). Using a threshold of 10 mut/Mb to distinguish “high” and “low” TMB status, the bTMB-tTMB pairs were classified into 4 categories: High-bTMB/High-tTMB, High-bTMB/Low-tTMB, Low-bTMB/High-tTMB, or Low-bTMB/Low-tTMB.

### Data Analysis

The following CPIs are represented in the time-to-treatment-failure (TTF) analysis: ipilimumab (Yervoy), nivolumab (Opdivo), pembrolizumab (Keytruda), atezolizumab (Tecentriq), avelumab (Bavencio), durvalumab (Imfinzi), cemiplimab (Libtayo), and dostarlimab (Jemperli). Time-to-treatment-failure was defined as the time from start of therapy to start of next therapy, death, or loss to follow up. Time-to-treatment-failure instances equal to zero days were excluded from analysis.

Statistical analyses were performed in R, a programming language and software environment for data science. The cor.test() function was used to calculate all Pearson’s correlation (*r*) values and the ccc() function (yardstick package) was used to calculate all Lin’s concordance (ρ) values. Hazard ratios (HRs) were computed with Cox regression (survival package). Ninety-five percent confidence intervals (CIs) were obtained by defining the range of the Cox regression coefficient as ±1.96SE.

This study has been determined to be exempt from IRB review by IntegReview IRB (Austin, Tx) according to 45 CFR 46.104.

## Results

### Discordance in Patient-Matched bTMB and tTMB Values

In alignment with previous studies,^18,20,21^ we observed patient-matched bTMB and tTMB values to be highly correlated (*r* = 0.61, *n* = 410, Fig. 1C). However, the absolute agreement—or concordance—between the 2 values was relatively low (ρ = 0.46, *n* = 410, Fig. 1C). This discordance resulted in conflicting “high” and “low” statuses in over one-third of cases when a threshold of 10 mut/Mb was applied, with 132 (32%) cases categorized as High-bTMB/Low-tTMB and 18 (4%) cases categorized as Low-bTMB/High-tTMB (*n* = 410, Fig. 1D). Approximately two-thirds of cases were in agreement, with 178 (44%) cases categorized as Low-bTMB/Low-tTMB and 82 (20%) cases categorized as High-bTMB/High-tTMB (*n* = 410, Fig. 1D).

Tumor mutation burden from blood consistently reported higher than from tissue in patient-matched specimens, with a median bTMB of 10.5 mut/Mb (interquartile range, IQR = 12.1 mut/Mb) and a median tTMB of 6.0 mut/Mb (IQR = 6.0 mut/Mb; *n* = 410; Fig. 1E). The median bTMB/tTMB ratio was 1.9 (IQR = 1.8, *n* = 410) and varied with tumor type, with significant differences between lung and colorectal, non-colorectal gastrointestinal, and breast cancers ([lung] *m* = 1.5, IQR = 1.3, *n* = 147; [colorectal] *m* = 2.5, IQR = 2.0, *n* = 44; [non-colorectal gastrointestinal] *m* = 2.9, IQR = 3.9, *n* = 38; [breast] *m* = 2.2, IQR = 2.2, *n* = 58; *P* < .05; Supplementary Fig. S1A). The concordance between bTMB-tTMB pairs was highest in gynecologic cancers and lowest in breast cancers ([gynecologic] ρ = 0.73, *n* = 22; [breast] ρ = 0.06, *n* = 58; Supplementary Fig. S1B).

### Variability in bTMB Among Blood-NGS Vendors

**Figure 2.**
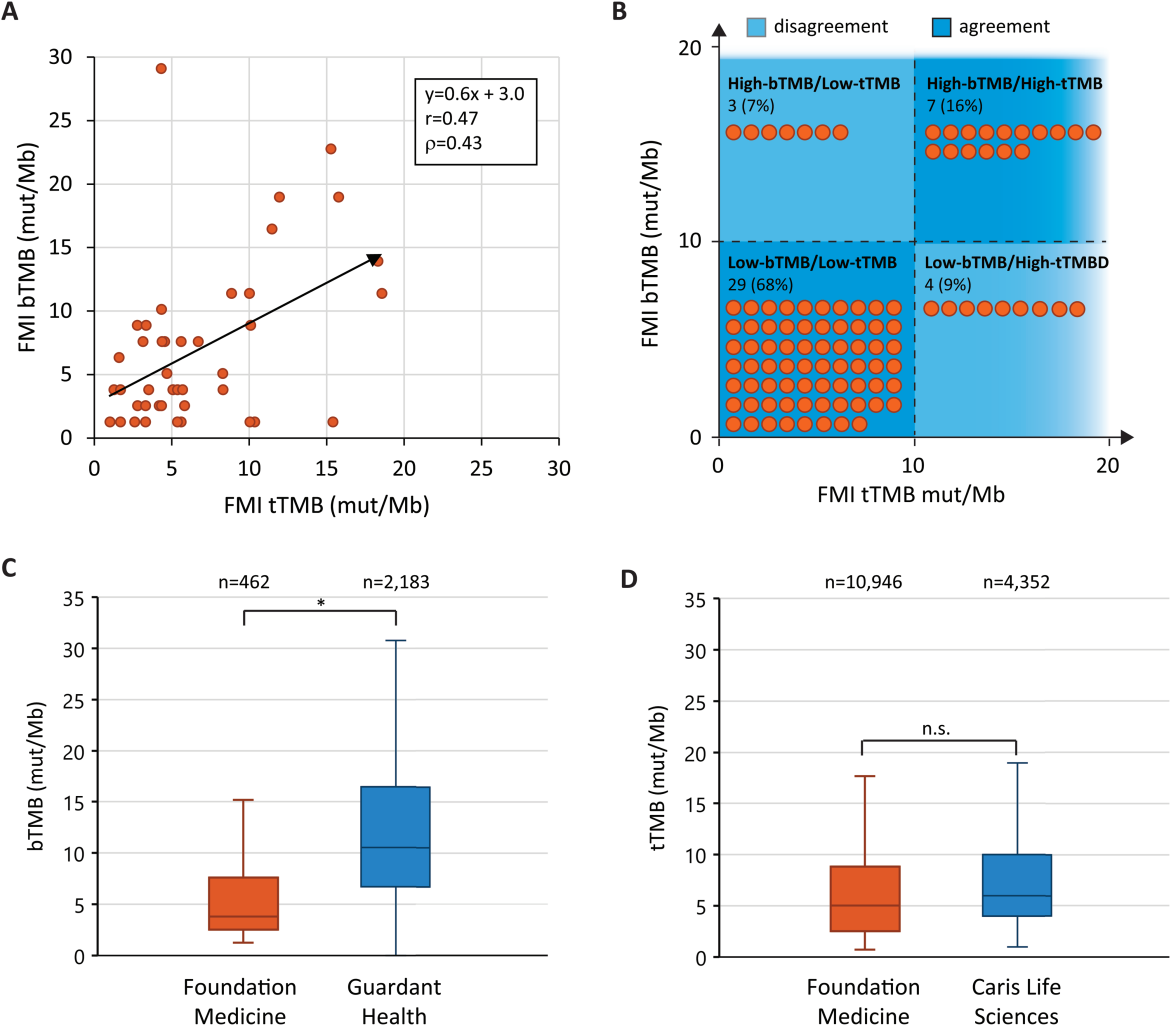
Variability in bTMB among blood-NGS vendors. (**A**) Subset of patient-matched bTMB-tTMB pairs wherein both blood- and tissue-NGS were performed by FMI. Random jitter was introduced on the *x*-axis to separate overlapping data points. Linear trendline, Pearson’s correlation coefficient (*r*), and Lin’s concordance coefficient (ρ) are shown. *n* = 42. (**B**) Distribution of FMI-only bTMB-tTMB pairs into high and low categorical statuses based on a threshold of 10 mut/Mb. Each orange circle represents 1% of bTMB-tTMB pairs. Quadrant color depicts agreement (dark blue) and disagreement (light blue) in bTMB and tTMB status. *n* = 42. (**C**) Distribution of total bTMB values by NGS vendor. GH: *m* = 10.5 mut/Mb; IQR = 9.8 mut/Mb; *n* = 2,183. FMI: *m* = 3.8 mut/Mb; IQR = 5.0 mut/Mb; *n* = 462. ∗p<.001. (**D**) Distribution of total tTMB values by NGS vendor. CLS: *m* = 6.0 mut/Mb; IQR = 6.0 mut/Mb; *n* = 4,353. FMI: *m* = 5.0 mut/Mb; IQR = 6.3 mut/Mb; *n* = 10,946. NGS, next-generation sequencing. m, median. IQR, interquartile range. Mut/Mb, mutations per megabase. GH, guardant health. FMI, Foundation Medicine. CLS, Caris Life Sciences. n.s., not significant.

The source of bTMB-tTMB discordance could be biological (ie, tumor heterogeneity) and/or technical (ie, vendor heterogeneity). Previous studies have demonstrated relatively strong agreement of bTMB-tTMB pairs reported by FMI (despite the plasma and tissue FMI assays having distinct technical and computational processes),^18,24^ so we next assessed the subset of 43 FMI-reported bTMB-tTMB pairs in our data set. While there was no improvement in the correlation or concordance of FMI-reported bTMB-tTMB pairs (*r* = .60, ρ = 0.58, *n* = 43, Fig. 2A), 84% of FMI-reported bTMB-tTMB pairs had agreement in “high” versus “low statuses (*n* = 43, Fig. 2b) when compared with 64% of vendor-mixed bTMB-tTMB pairs (410, Fig. 1D).

In assessing the FMI results, we noticed that the bTMB values were relatively low with 77% of FMI cases categorized as Low-bTMB (Fig. 2B) compared with just 48% of the vendor-mixed bTMB cases (Fig. 1D). We therefore next assessed the distribution of bTMB values by blood-NGS vendor. We included all available bTMB values regardless of whether a patient-matched tTMB value was available. Strikingly, GH reported significantly higher bTMB values than FMI with a median bTMB of 10.5 mut/mb (IQR = 9.8 mut/Mb, *n* = 2,183) from GH and 3.8 mut/Mb (IQR = 5.0 mut/Mb, *n* = 462) from FMI (*P* < .001, Fig. 2C). We performed a similar analysis with all tTMB values and found no significant differences between tissue-NGS vendors ([CLS] *m* = 6.0 mut/Mb, IQR = 6.0 mut/Mb, *n* = 4,352; [FMI] *m* = 5.0 mut/Mb, IQR = 6.3 mut/Mb, *n* = 10 946; Fig. 2D).

Besides heterogeneity in testing platforms, another source of bTMB-tTMB discordance could stem from disparate specimen collection dates, as extended periods of time can pass between specimen collections in real-world settings. This could allow for tumor evolution to confound results, especially as blood-NGS test results have proven sensitive to prior treatments and can be used to monitor for the emergence of drug-resistance alterations.^25-28^ Notably, we did not observe a correlation between bTMB/tTMB ratio and time between specimen collection, indicating that variability in specimen collection timing is not a major contributor to bTMB-tTMB discordance (*r* = –0.03, *n* = 410, Supplementary Fig. S2). A more in-depth analysis is required to understand how treatment influences the evolution of TMB.

### Differential Association of bTMB and tTMB with Immune Checkpoint Inhibition

**Figure 3.**
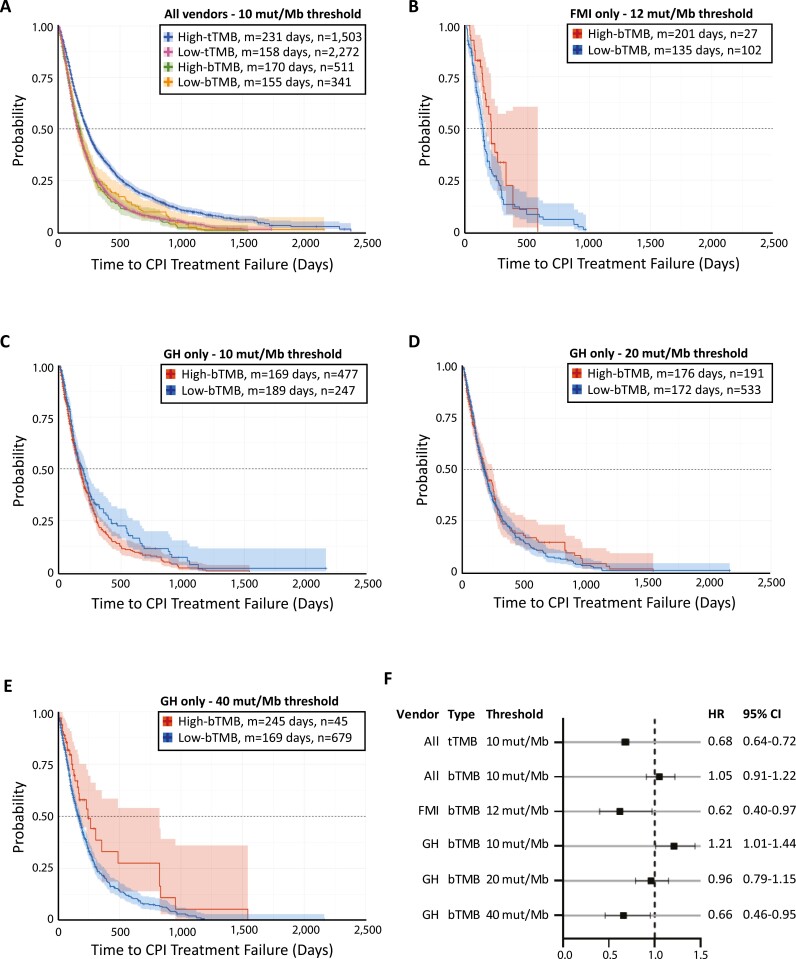
Differential association of bTMB and tTMB with immune checkpoint inhibition. Kaplan-Meier curves estimating CPI TTF in patients with TMB high and low results as defined by (**A**) tTMB ≥10 mut/Mb (blue), tTMB <10 mut/Mb (pink), bTMB ≥10 mut/Mb (green), and bTMB <10 mut/Mb (orange) for all vendors combined, (**B**) FMI-reported bTMB ≥12 mut/Mb (red) and <12 mut/Mb (blue), (**C**) GH-reported bTMB ≥10 mut/Mb (red) and <10 mut/Mb (blue), (**D**) GH-reported bTMB ≥20 mut/Mb (red) and <20 mut/Mb (blue), and (**E**) GH-reported bTMB ≥40 mut/Mb (red) and <40 mut/Mb (blue). Median values (m) and number of treatment instances (n) are shown. (**F**) HRs and 95% CIs for (A-E). CPI, immune checkpoint inhibitor. TTF, time to treatment failure. Mut/Mb, mutations per megabase. GH, Guardant Health. FMI, Foundation Medicine. m, median. HR, hazard ratio. CI, confidence interval.

We next assessed whether bTMB and tTMB are differentially associated with response to CPI therapy by measuring the TTF. As expected, tTMB associated with prolonged CPI TTF at a threshold of 10 mut/Mb (HR = 0.68; 95% CI = 0.64-0.72; *n* = 3,775) with a median of 231 days (High-tTMB) versus 158 days (Low-tTMB; Fig. 3A and F). This aligns with data from the KEYNOTE-158 trial wherein the median duration of exposure to pembrolizumab was 4.9 months (range: 0.03 to 35.2 months) in 105 patients with a tTMB ≥ 10 mut/Mb.^29^ In contrast, bTMB did not associate with prolonged CPI TTF at a threshold of 10 mut/Mb (HR = 1.05; 95% CI = 0.91-1.22; *n* = 852; Fig. 3A and F).

We next considered TMB results by vendor. tTMB from both FMI and CLS were associated with prolonged CPI TTF at a threshold of 10 mut/Mb ( Supplementary Fig. S3), although differences between the 2 vendors were apparent (Supplementary Fig. S3A asterisk). Interestingly, bTMB from FMI associated with prolonged CPI TTF at a threshold of 12 mut/Mb (HR = 0.62; 95% CI = 0.40-0.97; *n* = 129) with a median of 201 days (High-bTMB) versus 135 days (Low-bTMB; Fig. 3B and F). In contrast, bTMB from GH did not associate with prolonged CPI TTF at thresholds of 10 mut/Mb (Fig. 3C) or 20 mut/Mb (Fig. 3D), but did associate with prolonged CPI TTF at a threshold of 40 mut/Mb (HR = 0.66; 95% CI = 0.46-0.95; *n* = 724) with a median of 245 days (bTMB-High) versus 169 days (bTMB-Low; Fig. 3E and F). bTMB therefore required vendor-specific thresholds to associate with CPI benefit, with GH-reported values ≥40 mut/Mb resembling FMI-reported values ≥12 mut/Mb.

## Discussion

Tumor mutation burden is an evolving biomarker of CPI response. The tumor agnostic approval of pembrolizumab for High-TMB solid tumors was met with mixed responses from the scientific community, with critics arguing against a blanket 10 mut/Mb threshold to determine TMB “high” versus “low” status.^15,30,31^ Tumor mutation burden estimates are impacted by gene panel size, coverage/depth, and bioinformatics pipelines such that efforts to harmonize TMB reporting are needed to facilitate interpretation and decision making in clinical settings.^14,16^ In this study, we evaluate how the specimen (ie, blood versus tissue) used for TMB analysis affects outcomes in real-world settings and offer guidance for interpreting TMB results from various commercial tests.

Previous studies have noted a strong correlation between bTMB and tTMB in patients with cancer; however, these studies failed to evaluate overall concordance.^20,21^ We demonstrate herein that despite a strong correlation, bTMB typically reports higher than tTMB ultimately resulting in conflicting “high” versus “low” statuses in over one-third of patient cases wherein both blood- and tissue-NGS are performed (Fig. 1). These results align with several recent studies also demonstrating that bTMB reports higher than tTMB.^32,33^ Baden et al analyzed 344 patient-matched bTMB-tTMB pairs using the GH blood-NGS assay and FMI tissue-NGS assay, and noted a Spearman’s correlation of 0.56, a median bTMB of 13.5 mut/Mb, and a median tTMB of 7.7 mut/Mb.^32^ Drusbosky et al analyzed 5610 blood specimens using the GH blood NGS assay and reported that the 80th percentile bTMB was ≥16 mut/Mb tissue equivalency.^33^ The authors of the Drusbosky et al study concluded that blood-NGS may be particularly adept at estimating TMB because of its ability to sample tumor heterogeneity.^33^ While we agree that there may be important biological aspects contributing to the bTMB-tTMB discordance, our work reported herein specifically assesses vendor heterogeneity as a source of potential bTMB-tTMB discordance.

Our data demonstrate that bTMB values reported by GH tend to be higher than from FMI, with a median bTMB of 10.5 mut/Mb from GH versus 3.8 mut/Mb from FMI (Fig. 2C). Notably, a previous study by Qiu et al directly compared the GH and FMI blood-NGS assays with replicate blood samples and found bTMB from GH to be higher than from FMI (the authors did not perform a statistical comparison).^20^ There are several technical factors that may account for this vendor variability. While both GH and FMI filter out potential germline and oncogenic driver alterations, as well as those associated with clonal hematopoiesis, GH counts both single-nucleotide variants (SNVs) and short insertions/deletions (INDELs) while FMI only counts SNVs.^18,21,23^ Furthermore, GH counts variants at all allele frequencies while FMI only counts variants at ≥0.5% allele frequency.^18,21,23^ Further understanding of these and other sources of variability will help efforts to harmonize TMB estimation among NGS vendors.

Ultimately, the variability in TMB estimation across NGS vendors demonstrated herein challenges the notion that a blanket 10 mut/MB threshold can successfully identify patients who may benefit from immune checkpoint inhibition. We demonstrate that bTMB from both GH and FMI are associated with response to CPI therapy, albeit at different thresholds (Fig. 3), thereby showcasing the importance of considering both tumor and assay heterogeneity when interpreting molecular results. We argue that separate “high” versus “low” TMB thresholds should be considered across various blood-NGS testing platforms and caution clinicians against extending the 10 mut/Mb threshold from the FMI tissue CDx^15^ to other NGS assays. An important aim of future studies will be to assess bTMB and tTMB within specific tumor types with a particular focus on those with broad CPI usage.

## Supplementary Material

oyab064_suppl_Supplementary_Figure_S1

oyab064_suppl_Supplementary_Figure_S2

oyab064_suppl_Supplementary_Figure_S3

## Data Availability

The data underlying this article will be shared on reasonable request to the corresponding author.
