# Supplementary figures and images for: Discordance in Tumor Mutation Burden from Blood and Tissue Affects Association with Response to Immune Checkpoint Inhibition in Real-World Settings
Source: Oncologist. 2022 Feb 19;27(3):175–82. doi: 10.1093/oncolo/oyab064 (PMC8914506; doi:10.1093/oncolo/oyab064)

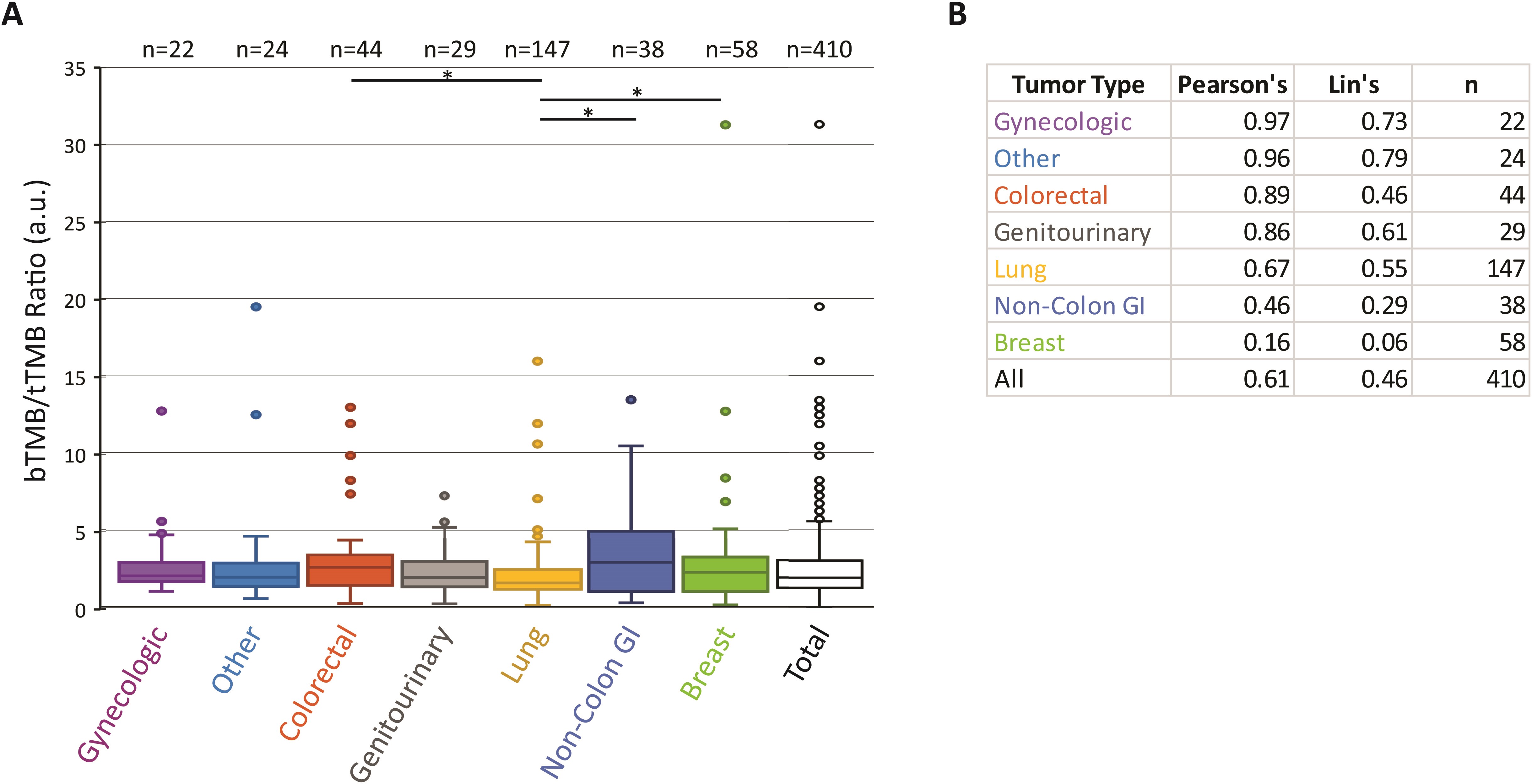

Supplement: oyab064_suppl_Supplementary_Figure_S1 [file oyab064_suppl_supplementary_figure_s1.jpeg]

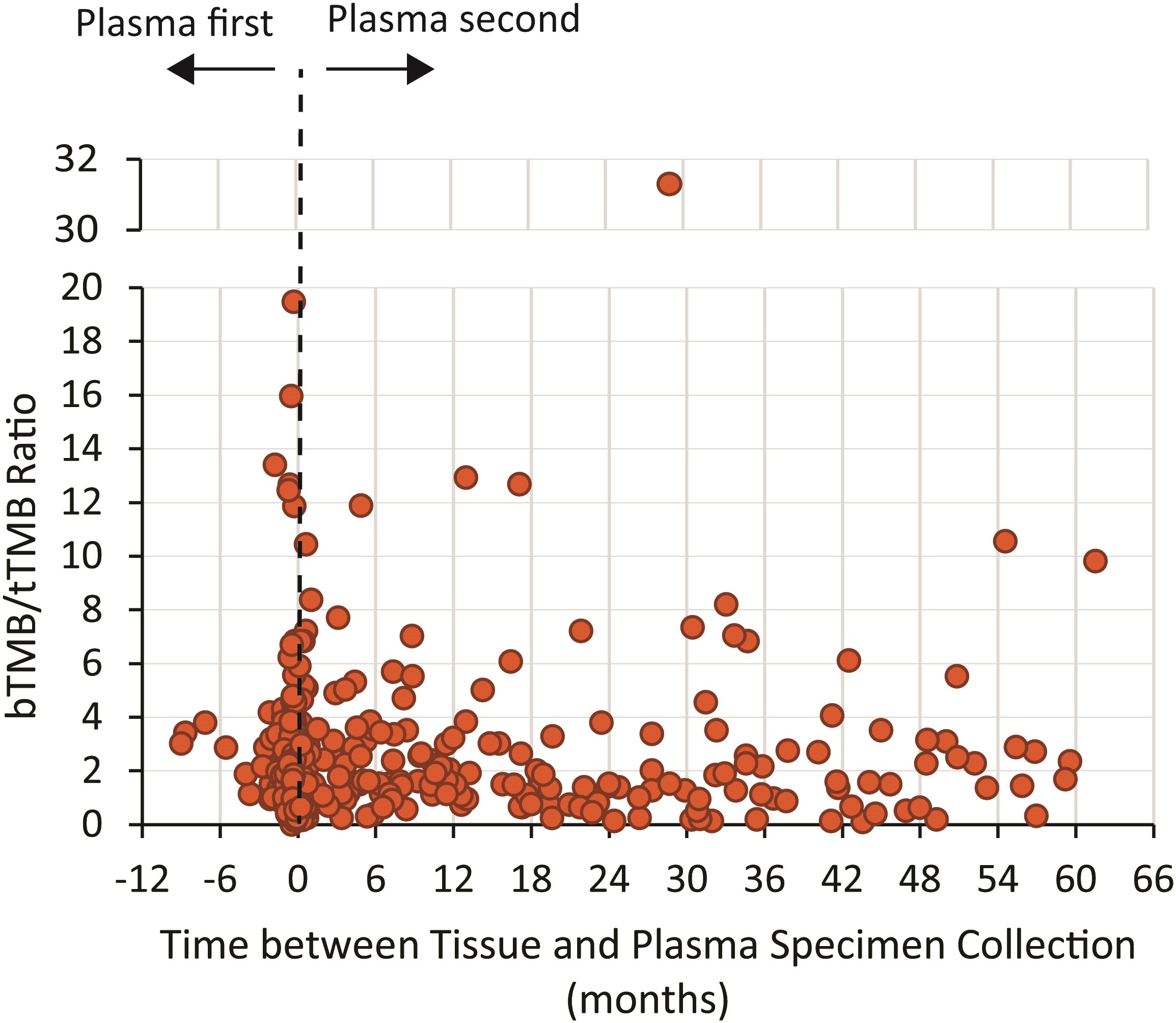

Supplement: oyab064_suppl_Supplementary_Figure_S2 [file oyab064_suppl_supplementary_figure_s2.jpeg]

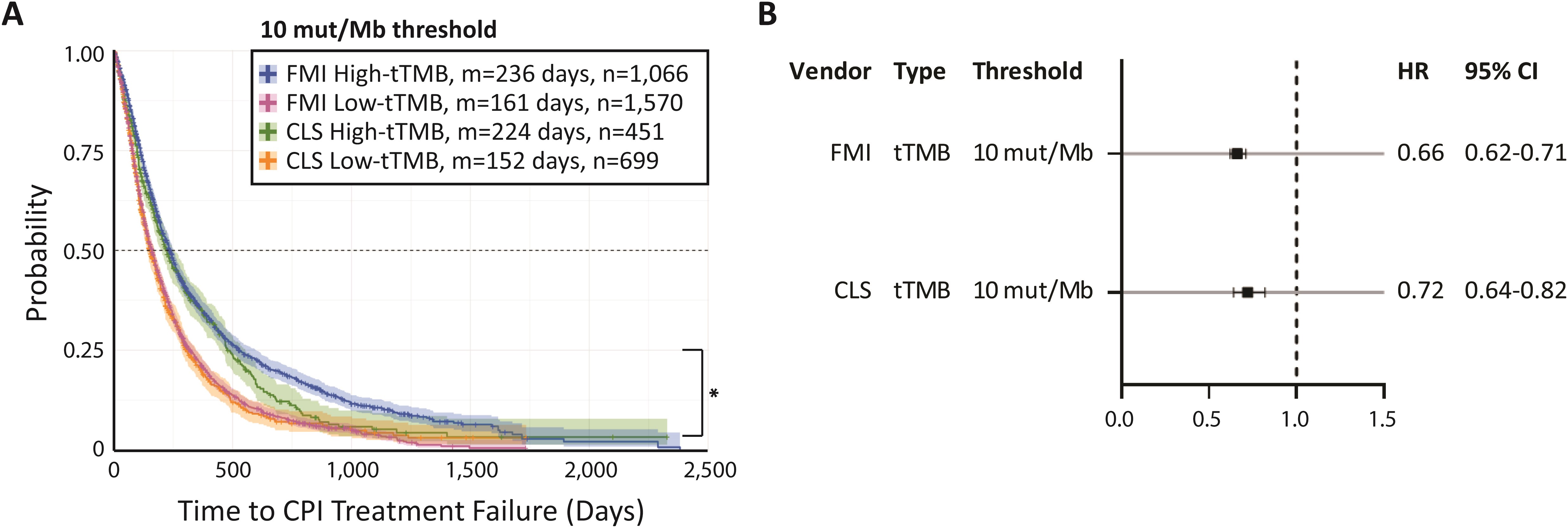

Supplement: oyab064_suppl_Supplementary_Figure_S3 [file oyab064_suppl_supplementary_figure_s3.jpeg]
